# NK cells encapsulated in micro/macropore-forming hydrogels via 3D bioprinting for tumor immunotherapy

**DOI:** 10.1186/s40824-023-00403-9

**Published:** 2023-06-22

**Authors:** Dahong Kim, Seona Jo, Dongjin Lee, Seok-Min Kim, Ji Min Seok, Seon Ju Yeo, Jun Hee Lee, Jae Jong Lee, Kangwon Lee, Tae-Don Kim, Su A Park

**Affiliations:** 1 Nano Convergence & Manufacturing Systems, Korea Institute of Machinery and Materials (KIMM), Daejeon, 34103 Republic of Korea; 2 Department of Applied Bioengineering, Graduate School of Convergence Science and Technology, Seoul National University, Seoul, 08826 Republic of Korea; 3 Immunotherapy Research Center, Korea Research Institute of Bioscience and Biotechnology (KRIBB), Daejeon, 34141 Republic of Korea; 4 Department of Functional Genomics, KRIBB School of Bioscience, Korea University of Science and Technology (UST), Daejeon, 34113 Republic of Korea; 5 Research Institute for Convergence Science, Seoul National University, Seoul, 08826 Republic of Korea; 6 Biomedical Mathematics Group, Institute for Basic Science, Daejeon, 34126 Republic of Korea; 7 Department of Biopharmaceutical Convergence, School of Pharmacy, Sungkyunkwan University, Suwon, 16419 Republic of Korea

**Keywords:** Solid tumor, Immunotherapy, NK cells, Micro/macropore-forming, 3D bioprinting

## Abstract

**Background:**

Patients face a serious threat if a solid tumor leaves behind partial residuals or cannot be completely removed after surgical resection. Immunotherapy has attracted attention as a method to prevent this condition. However, the conventional immunotherapy method targeting solid tumors, that is, intravenous injection, has limitations in homing in on the tumor and in vivo expansion and has not shown effective clinical results.

**Method:**

To overcome these limitations, NK cells (Natural killer cells) were encapsulated in micro/macropore-forming hydrogels using 3D bioprinting to target solid tumors. Sodium alginate and gelatin were used to prepare micro-macroporous hydrogels. The gelatin contained in the alginate hydrogel was removed because of the thermal sensitivity of the gelatin, which can generate interconnected micropores where the gelatin was released. Therefore, macropores can be formed through bioprinting and micropores can be formed using thermally sensitive gelatin to make macroporous hydrogels.

**Results:**

It was confirmed that intentionally formed micropores could help NK cells to aggregate easily, which enhances cell viability, lysis activity, and cytokine release. Macropores can be formed using 3D bioprinting, which enables NK cells to receive the essential elements. We also characterized the functionality of NK 92 and zEGFR-CAR-NK cells in the pore-forming hydrogel. The antitumor effects on leukemia and solid tumors were investigated using an in vitro model.

**Conclusion:**

We demonstrated that the hydrogel encapsulating NK cells created an appropriate micro–macro environment for clinical applications of NK cell therapy for both leukemia and solid tumors via 3D bioprinting. 3D bioprinting makes macro-scale clinical applications possible, and the automatic process shows potential for development as an off-the-shelf immunotherapy product. This immunotherapy system could provide a clinical option for preventing tumor relapse and metastasis after tumor resection.

**Graphical Abstract:**

Micro/macropore-forming hydrogel with NK cells fabricated by 3D bioprinting and implanted into the tumor site.

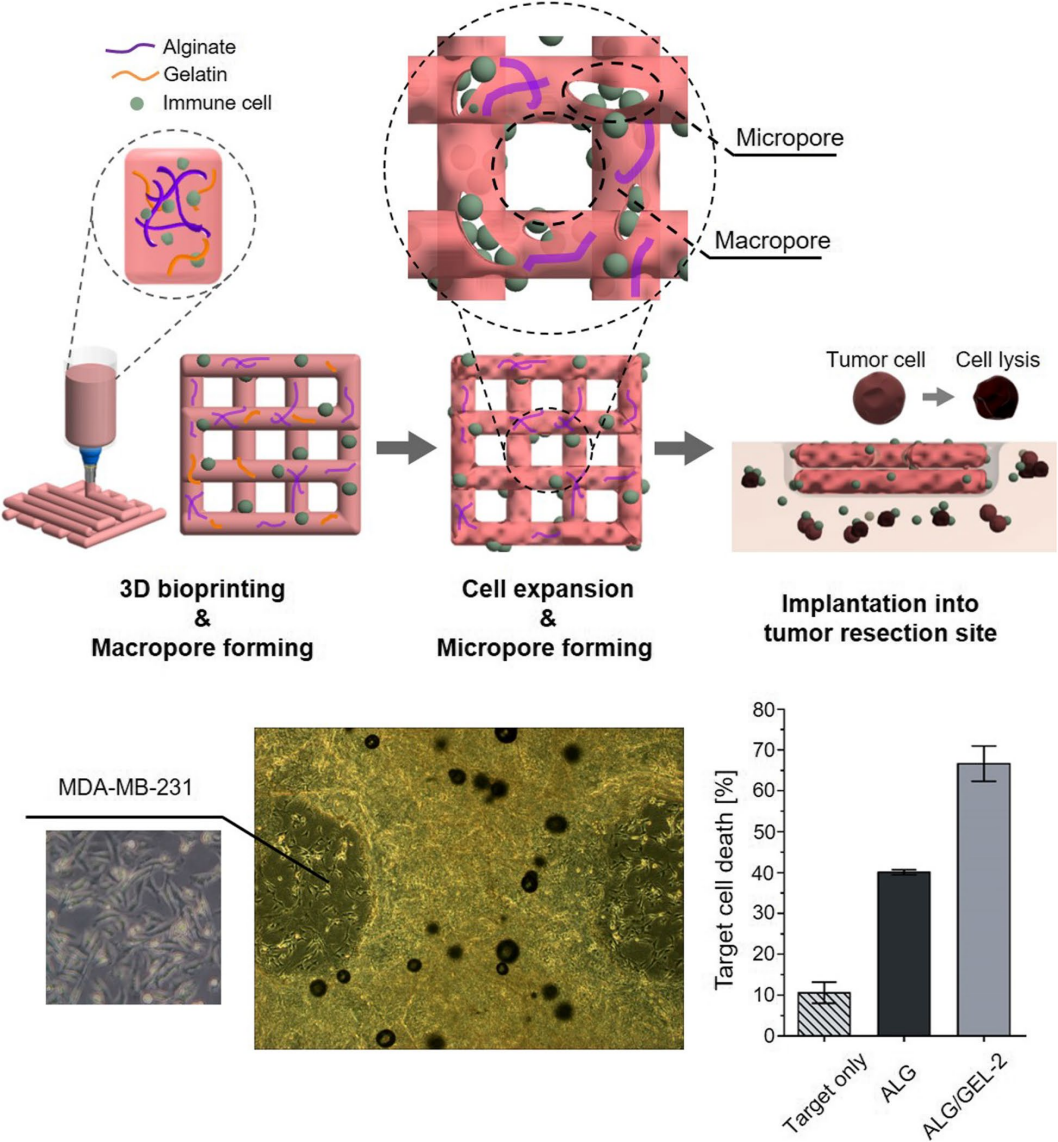

**Supplementary Information:**

The online version contains supplementary material available at 10.1186/s40824-023-00403-9.

## Introduction

Solid tumors are treated through surgical resection, but they are considered a major threat to the life of a patient when they leave behind partial residuals or cannot be completely removed owing to large defects in the surgical site. Malignant cells continue to form an immunosuppressive tumor microenvironment (TME) and move toward survival [1–3]. Strategies such as radiotherapy and chemotherapy are widely used to eliminate resistant tumor cells. Immunotherapy, a cancer therapy that involves or uses components of the immune system, is emerging as an effective treatment method [4–7].

Immunotherapy has been developed for cancer treatment, including cancer vaccines, cytokine therapies, immune checkpoint inhibitors, and adoptive cell transfer. To enhance an existing immune response, adoptive cell transfer, in which leukocytes such as macrophages and T or NK cells are extracted from peripheral blood and reintroduced into the patient, has been exploited [7–12]. In particular, NK cell-based immunotherapy has attracted attention because it is relatively safe regarding critical side effects (for example, cytokine release syndrome, neurotoxicity, and on-target off-tumor effects) and rarely causes graft-versus-host disease. Owing to these advantages, it shows potential as an off-the-shelf product for immediate clinical use [8, 13–15].

However, there are some limitations to using NK cells for immunotherapy [15–17]. Intravenous injection is widely used for immune cell transfer, but the results shown in clinical trials are not particularly good for solid tumors. The two main obstacles are the high expansion rate of immune cells with suitable viability and targeting effect, homing to the tumor site, and activity in the TME that results in the loss of long-term anti-tumor efficiency [4, 6, 13, 17, 18]. To overcome these issues, various cell culture methods were needed to increase the viability and activity of NK cells, including 3D bioprinting.

Various efforts have focused on enhancing the functions of immune cells using 3D culture systems and driving immune cells to the tumor site. The generation of porous microspheres with encapsulated NK-92MI cells effectively killed tumors through in situ injection [19]. To develop a scale-up method, other researchers fabricated a dual-layered hydrogel/microsphere in which many cell-laden microspheres were encapsulated in the hydrogel [20]. A hyaluronic acid-based niche scaffold with NK92 cell lines and human EGFR (epidermal growth factor receptor)-specific CAR-NK (chimeric antigen-receptor-modified Natural killer) cells were used to prevent relapse and metastases after tumor resection [18].

3D bioprinting is suitable for creating 3D culture systems and macroscale clinical applications. In addition, it can print structures that are suitable for insertion into tumor resection sites. By forming macropores through 3D bioprinting, it is possible to transport oxygen, nutrients, and IL-2 cytokines, which are essential for cell survival and expansion [21, 22]. 3D bioprinting has potential as an off-the-shelf product platform because its automatic processing is faster than hanging drop or other seeding methods. Micro/macropore-forming hydrogels with the NK92 cell line and human EGFR-specific CAR-NK cells were fabricated by 3D bioprinting for cell-based immunotherapy. Pore-forming hydrogels can offer an environment that facilitates the easy aggregation of NK cells, which is directly related to viability, proliferation, and activities by cell-to-cell interaction [23, 24]. In addition, the extracellular matrix (ECM)-like structure of hydrogels can help in enduring the harsh conditions of the TME and enhance NK cell activities such as cytotoxicity and cytokine release by 3D cultures [18, 25, 26]. By using 3D bioprinting, macropores can be formed and automatic processing for off-the-shelf product platforms can be achieved. Micro/macropore-forming hydrogels with NK cells fabricated by 3D bioprinting enable NK cell-based immunotherapy to directly interact with residual tumors to prevent recurrence and metastasis after tumor resection.

In this study, we investigated the antitumor effect of NK cells encapsulated in micro/macropore-forming hydrogels using 3D bioprinting for tumor immunotherapy. This hydrogel was made from alginate and gelatin, which are naturally derived hydrogels that can be formed by using the thermosensitive property of gelatin [27]. The hydrogel optimized the printability and viability of NK cells using the alginate to gelation ratio. We also characterized the micro/macropore-forming hydrogel and the functionality of NK 92 and zEGFR-CAR-NK cells in the pore-forming hydrogel. The antitumor effects on leukemia and solid tumors were investigated using an in vitro model. We demonstrated that the hydrogel encapsulating NK cells created an appropriate micro–macro environment for clinical applications of NK cell therapy for both leukemia and solid tumors via 3D bioprinting.

## Materials and methods

### Preparation of cell-laden micro/macroporous hydrogel

**Fig. 1:**
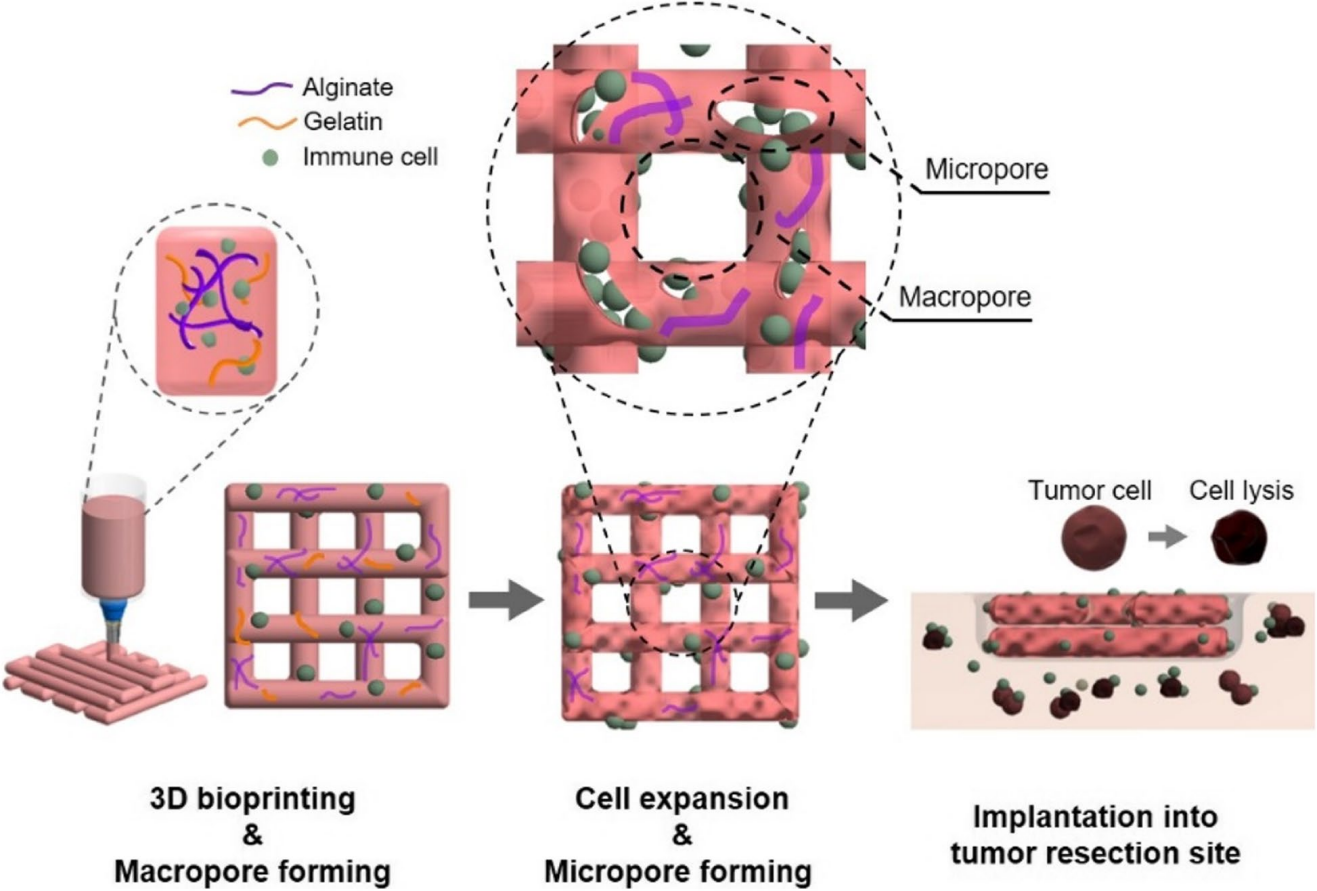
Schematic illustration of micro/macropore-forming hydrogel with NK cells fabricated by 3D bioprinting and implanted into the tumor site

An overview of the fabrication process of the porous hydrogel is presented (Fig. 1). Sodium alginate and gelatin were used to prepare micro-macroporous hydrogels. Sodium alginate powder was dissolved in alpha-MEM so that the total weight percent was 3 wt.% alginate hydrogel for characterization of the hydrogel. In addition, 10 wt.% gelatin were separately dissolved in alpha-mem media at 50 ℃. The gelatin solution was blended with alginate according to the weight ratio shown in Table 1, and NK92 or zEGFR-CAR-NK cells were added and blended manually into the mixture. NK92 cells were prepared 4 × 10^5^ per well (cell/complete media: 2 × 10^5^/mL) in 12 well plates to compare to the 2D cultured group (Supplementary Fig. 1), and zEGFR-CAR-NK cells were prepared 2.5 × 10^5^ per well (cell/complete media: 2.5 × 10^5^/mL) in 24 well plates for the anti-tumor effect of pore-forming hydrogel with CAR-NK cells. Then, 1 wt.% CaCl_2_ dissolved in filtered phosphate buffered saline (PBS) was added to the blended alginate-gelatin hydrogel to pre-crosslink the sodium alginate. The mixtures were manually mixed evenly using a spatula and loaded into a syringe for printing. The macroporous structure was printed using 3D bioprinting (PROTEK, Korea Institute of Machinery and Materials, Korea). Each group was controlled by a proper pressure and feed rate (Supplementary Fig. 2). After printing, the alginate-gelatin hydrogels were put into the 3% CaCl_2_ solution for 1 min for physical crosslinking sodium alginate only. The number of cells encapsulated in the cell-laden hydrogel was 4 × 10^6^ cells per well. The printed hydrogels were incubated in 12 well plates filled with 2 mL complete media at 37 °C. Then, the gelatin contained in the alginate hydrogel was removed because of the thermal sensitivity of the gelatin, which can generate interconnected micropores where the gelatin was released. Therefore, macropores can be formed through bioprinting and micropores can be formed using thermally sensitive gelatin to make macroporous hydrogels.

**Table 1:** The weight ratio of alginate to gelatin in bioink formulation

Group name	Alginate [%]	Gelatin [%]	Ratio
ALG	3.0	0.0	1:0
ALG/GEL-1	3.0	0.6	5:1
ALG/GEL-2	3.0	1.0	3:1
ALG/GEL-3	3.0	3.0	1:1

### Characterization of pore-forming hydrogel

The rheological behavior of the hydrogels was investigated using a rheometer (AR2000ex, TA Instruments, USA) with a 40 mm aluminum plate as the parallel plate. The samples were placed on the bottom plate and the parallel plate geometry was fitted. Amplitude sweeps were performed at 25 °C.

The chemical composition of the porous hydrogel was analyzed using Fourier-transform infrared spectroscopy (FTIR; NICOLET 6700; Thermo Fisher Scientific, USA) with 4 cm^−1^ resolutions. All samples were lyophilized after gelatin release, with the exception of the gelatin group.

### Structure and printability/shrinkage of the porous hydrogel construct

A scanning electron microscope (Sirion; FEI, USA) was used to investigate the pore structure of the printed hydrogel before and after gelatin release. The printed alginate-gelatin hydrogels were lyophilized at -85 ℃ for 3 d using a freeze-dryer (Il Shin Biobase Co. Ltd., Korea). The dried hydrogels were coated with gold for 3 min at 30 mA using a sputter coater (SCD 005; BAL-TEC, Liechtenstein).

The average printability and shrinkage were measured using ImageJ software (National Institutes of Health) at nine different points per sample. Printability was calculated using semi-quantification of printability [28]. The degree of bioink gelation was determined through the bioink printability (Pr) based on the circularity (C) and square shape.

The printability of the hydrogels was calculated using the following equation: Pr = (*π/4)∙(1/C)* = *L*^*2*^*/16A*.

*L* means perimeter and *A* means area of printed structure. The interconnecting channels of the constructs would exhibit square shape for an optimum gelation condition or perfect printability state, and the Pr value was 1.

The ratio of alginate to gelatin was different for each group, and only alginate was physically crosslinked by CaCl_2_; therefore, the degree of shrinkage was different. A 12 mm × 12 mm (W × D) structure was printed, and its length was measured after crosslinking in 3% CaCl_2_ for 1 min. The shrinkage was calculated using five points per sample for the four samples. The percentage shrinkage was calculated using the following formula: (L_after_ / L_before_) × 100. The length of the hydrogel was measured immediately after printing (L_before_). Then, the length of the hydrogel after crosslinking (L_after_) was measured, and the ratio of L_before_ and L_after_ was calculated.

### Cell culture

The NK92 (human natural killer cell line (ATCC CRL-2407TM) and K562 (human leukemia cell line, ATCC CCL-86TM) were purchased from the American Type Culture Collection (Manassas, VA, USA). NK92 cells and zEGFR-CAR-NK cells were cultured in alpha minimum essential medium containing 12.5% fetal bovine serum (FBS), 12.5% horse serum, and 1% anti-anti. To prepare the complete medium, it was supplemented with 0.2 mM inositol, 0.1 mM 2-mercaptoethanol, 0.02 mM folic acid, and 200 U/ml recombinant IL-2 (Peprotech, Cat. 200–02). K562 cell lines were grown in Roswell Park Memorial Institute Medium (RPMI) 1640 medium containing 10% FBS and 1% anti-anti.

### Evaluation of NK92 cell viability and proliferation assay in the porous hydrogel

A live and dead kit (LIVE/DEAD™ Viability/Cytotoxicity Kit; Invitrogen, USA) was used to assess the viability of NK92 cells in the hydrogel. Calcein-AM (2 µL) and ethidium homodimer-1 (8 µL) were added to 10 mL PBS to prepare the staining solution. After removing the medium, the hydrogel groups were washed with PBS, and 1 mL of the staining solution was added to the hydrogel encapsulating NK92 cells. After 1 h of incubation, the fluorescence of NK92 cells was investigated using a fluorescence microscope (Eclipse Ti; Nikon, Japan). The viability of NK cells after 1, 4, and 7 d was confirmed. In addition, WST-1 (Premix WST-1 Cell Proliferation Assay System; Takara, Japan) was used to measure mitochondrial dehydrogenase activity during the proliferation of NK92 cells in the hydrogel. A reagent was prepared by mixing the WST-1 solution and PBS at a ratio of 10:1, and absorbance was measured using a microplate reader (SpectraMax iD3; Molecular Devices, USA).

### In vitro cytotoxicity and cytokine releasing

To investigate the functionality of NK92 cells encapsulated in a porous hydrogel, crosslinked alginate in the hydrogel was degraded using 8 mM Ethylenediaminetetraaceticacid (EDTA) (Supplementary Fig. 3) [29]. The hydrogel was incubated for 30 min and centrifuged to obtain 3D cultured cells. The cytotoxicity was investigated using a calcein-AM-based assay. Target cancer cells were stained with calcein-AM (Invitrogen, USA) for 1 h at 37 °C. The labeled target cells (1 × 10^4^ cells / 100 µL) and NK92 cells were placed in 96-well round-bottom plates at effector-to-target (E/T) ratios of 2:1 and 1:1 for 4 h. The maximum release was measured by adding 4% Triton X-100 to the target cell. The percentage cytotoxicity was calculated using the following formula: (sample release – spontaneous release) / (maximum release – spontaneous release) × 100.

Cytokine release, which affects the target, was measured using an ELISA. The supernatant of the tumor cell media co-cultured with NK cells was collected, and the concentration of released cytokines (interferon gamma, TNF-alpha) was investigated. An ELISA kit (Human IFN gamma uncoated ELISA, Human TNF alpha uncoated ELISA; Invitrogen, USA) was used according to the instructions of the manufacturer.

### Flow cytometry

The cells were washed with FACS buffer (PBS supplemented with 0.1% FBS and 0.4% EDTA) and centrifuged to obtain cell pellets. The cell pellets were stained with antibodies for 30 min and analyzed by fluorescence-activated cell sorting (FACS). For the NK cell apoptosis assay, APC-conjugated annexin V and 7-AAD solution were added to the samples in 100 µL binding buffer at RT for 15 min. After staining, binding buffer was added to the sample tube and apoptosis was analyzed by flow cytometry.

### Real-time PCR

Ribonucleic acid (RNA) was extracted by synthesis using a complementary deoxyribonucleic acid (cDNA) kit (Toyobo, Japan). The study was compliant with the guidelines of the Korea Research Institute of Bioscience and Biotechnology (KRIBB). Real-time PCR was performed using a Thermal Cycler Dice TP800 with a qPCR Master Mix (SFC). All data were normalized to the housekeeping gene GAPDH. Primer sequences for the genes are listed in Supplementary Table 1.

### Cytotoxic effect of pore-forming hydrogel

To confirm the tumor cytotoxic effect when the hydrogel was naturally degraded to mimic the hydrogel inserted into the tumor site, the following method was used. The cell-laden hydrogel crosslinked with 3% CaCl_2_ was subjected to cell expansion for 5 d. The cell number of the encapsulating pore-forming hydrogel was 2.5 × 10^5^ per well. MDA-MB-231 cells were labeled using CellTrace (CellTrace™ Violet Cell Proliferation Kit; Thermo Fisher, USA) to distinguish the target cells from zEGFR-CAR-NK cells. Target cells were adhered to 2.5 × 10^5^ cells per 500 µL of RPMI medium in 24 well plates and co-cultured with cell-laden hydrogel for 24 h. After the co-culture, the cell-laden hydrogel was removed. To analyze target cell death, only cells stained with 7-AAD were analyzed by flow cytometry.

### Statistical analysis

All quantitative data are presented as mean ± standard deviation. The results were analyzed using one-way analysis of variance followed by Tukey’s post-hoc test. A *p*-value < 0.05 was considered significant and indicated by asterisk in the figures. Statistical analysis was conducted using Origin software (Origin 8.6 ver., OriginLab Corporation, USA).

## Results

### Characterization of micro/macropore-forming hydrogel

The viability and proliferation of NK cells are promoted by cell–cell interactions while NK cells aggregate [23, 24]. To expand the cells in the hydrogel, the transport of oxygen, nutrients, and cytokines must be facilitated. An environment that helps cell aggregation through micropores and transport through macropores can be offered to enhance the viability and functionality of NK cells.

Bio-inks containing alginate and gelatin are required to fabricate micro/macropore hydrogels by 3D bioprinting. Micropores are controlled by the ratio of alginate to gelatin. Because gelatin is a thermally sensitive hydrogel, micropores are formed as it is released during incubation [27, 30]. Macroporous structures were fabricated using 3D bioprinting.

**Fig. 2:**
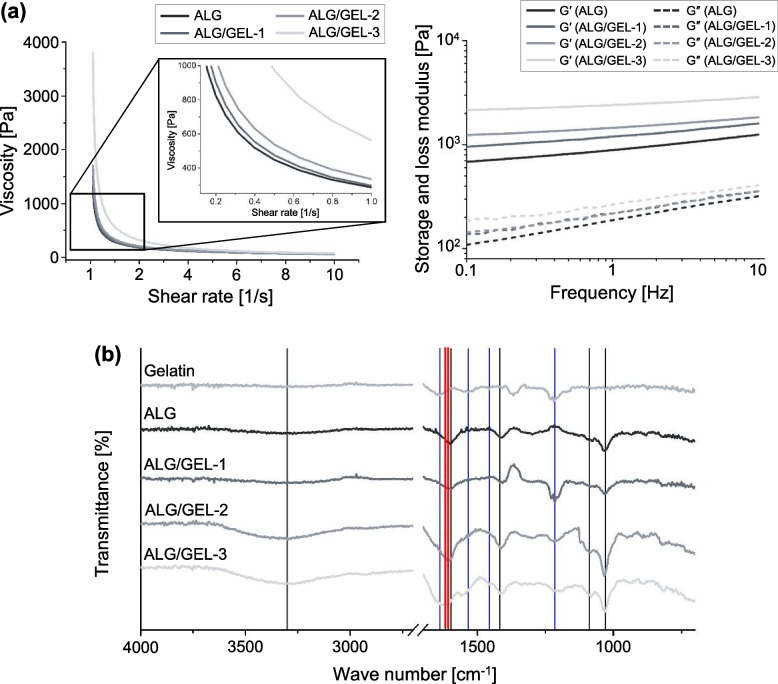
Characterization of micro/macropore-forming hydrogel for 3D bioprinting. **a** Viscosity and rheological properties of bioink. **b** Chemical construction of bioink and gelatin

Rheological tests were performed to investigate the viscoelastic properties of the bioinks, which are known to affect printability and cell viability during printing [28, 31, 32]. All groups showed that the viscosity decreased with increasing shear rate, that is, the shear-thinning properties (Fig. 2a). When the hydrogel was extruded from the nozzle, its viscosity decreased with increasing shear stress, resulting in hydrogel formation [33]. Thus, the cell could be subjected to lower shear stress [34]. In addition, as the gelatin ratio increased, viscosity increased. In the results of the storage and loss modulus, G’ was higher than G’’ in all groups, indicating that the elastic behavior of the bioink was dominant (Fig. 2b). The higher the proportion of gelatin, the more solid-like the behavior, which requires high pneumatic pressure for printing (Supplementary Fig. 1).

The molecular interactions of the pore-forming hydrogels were investigated using FT-IR spectroscopy (Fig. 2c). Only the alginate group (ALG) showed peaks at 3300 cm^−1^ from the stretching peak of O–H, 1569 and 1417 cm^−1^ from asymmetric and symmetric stretching vibrations of COO, respectively [35]. In addition, the C–C vibration of the M block and C-O–H stretching vibration of the G block in alginate appeared at 1089 and 1029 cm^−1^, respectively [35, 36]. Pure gelatin showed amide acids at 1637, 1533, and 1216 cm^−1^, indicating amide 1 (C = O and C-N stretching vibrations), amide 2 (N–H bending vibration and C-N stretching vibration), and amide 3 (N–H bending vibration and C-N stretching vibration), respectively. The bands around 3300 and 1456 result from the O–H stretching and C-H bending vibrations, respectively [37, 38]. A characteristic change was observed in the groups to which gelatin was added. The peak of the carboxyl groups in alginate, 1596, shifted from 1607 to 1616 cm^−1^. This result shows the electrostatic interaction between the carboxyl groups of alginate and the amino groups of gelatin [39].

### Bioprinted micro/macroporous hydrogel

**Fig. 3:**
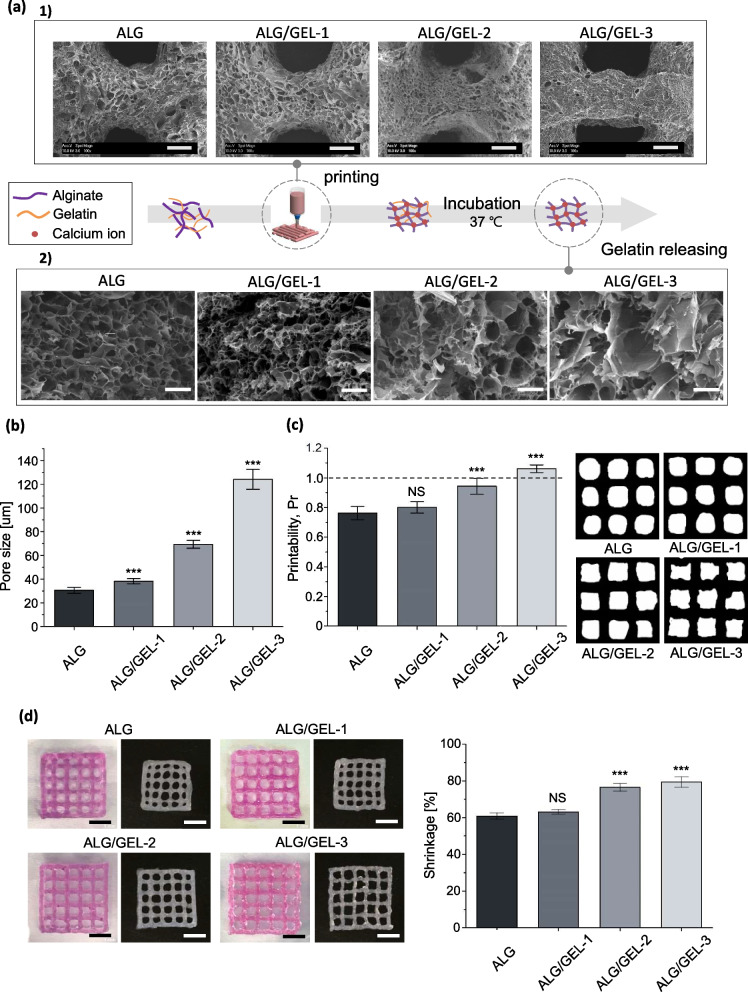
Bioprinted micro/macroporous hydrogel. **a** Representative SEM image of printed pore-forming hydrogel. 1) The pore structure immediately after printing, scale bar = 500 μm. 2) and after incubation for 1 day (after releasing the gelatin), scale bar = 100 μm. **b** Micro pore size of the pore-forming hydrogel. **c** Semi-quantification of printability. **d** Shrinkage rate of hydrogel, scale bar = 5 mm. [*n* = 4, NS: no significant difference, ***p < 0.001]

The structure of the micropores was investigated immediately after printing and after incubation for 1 d (Fig. 3a). The structure of the hydrogel immediately after printing according to the proportion of gelatin is shown (Fig. 3a 1)). In this process, the higher the percentage of gelatin, the smaller the pore size. The alginate and gelatin networks were narrow owing to electrostatic interactions between the carboxyl groups of alginate and the amino groups of gelatin. The pore structure and pore size after alginate was only crosslinked by calcium chloride (CaCl_2_), and gelatin was released from the hydrogel (Fig. 3b-c). As the gelatin ratio increased, the micropore size also tended to increase. The pore size of ALG was 30.54 μm, ALG/GEL-1 was 38.26 μm, ALG/GEL-2 was 69.33 μm, and ALG/GEL-3 was 124.03 μm. These results confirmed that the amount of gelatin contained in the hydrogel could form micropores and adjust their size. This enables NK cells to form cell clusters, providing an environment favorable for cell viability and proliferation.

Printability is important for accurately realizing the macropore structure and fabricating a hydrogel by simulating the boundary after tumor resection. In addition, rapid shrinkage of the hydrogel network by crosslinking affects the cell viability [31, 40]. Semi-quantification of printability is shown (Fig. 3d). The closer it is to a perfect square shape, the closer the number is to one. In general, a liquid-like extrusion forms a circular shape, and the Pr value is smaller than 1. Conversely, a solid-like hydrogel forms a rough shape and the Pr value is greater than 1 [28]. Hydrogels with ideal printability were confirmed based on the gelatin ratio of the bioink. ALG/GEL-2 (0.95 ± 0.053) and ALG/GEL-3 (1.06 ± 0.026) had values close to 1. The viscosity and elastic properties were increased by gelatin, which were close to the ideal printability value [34, 39]. Sudden contraction of the cell environment affects cell viability [31]. Therefore, shrinkage was investigated with respect to the ratio before and after hydrogel shrinkage. The higher the proportion of gelatin, the lower the shrinkage that tended to occur [39]. This is more likely to be advantageous for cell viability in the group with less shrinkage.

### Viability and proliferation of encapsulated NK92 cells in the pore-forming hydrogel

**Fig. 4:**
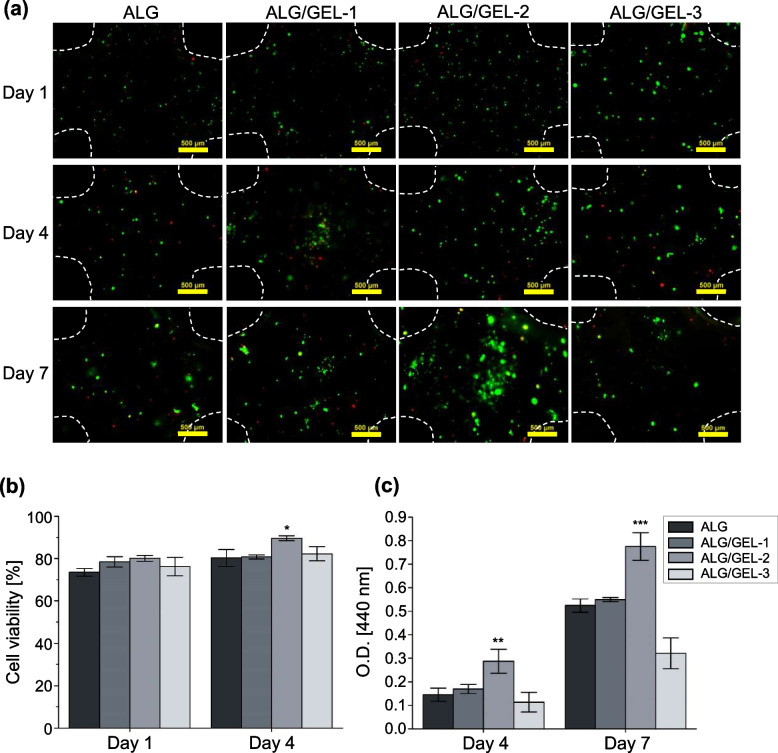
Viability and proliferation of encapsulated NK92 in the pore-forming hydrogel. **a** Live and dead assay of NK92 cells encapsulated in alginate and pore-forming hydrogels on day 1, 4, and 7 after printing, scale bar = 500 μm. **b** Cell viability from live and dead assay. **c** Cell proliferation of NK92 cell. [*n* = 4, NS: no significant difference, *p < 0.05, **p < 0.01 and ***p < 0.001]

The cell viability and proliferation of each pore-forming hydrogel were investigated using live and dead assays and WST-1, respectively. On day 1, it was confirmed that the cell distribution was relatively uniform, and cell aggregation started partially after day 4 in the three groups containing gelatin. On day 7, cell aggregation was active in ALG/GEL-2, and in particular, proliferation was higher than that in ALG (approximately 1.48 fold) (Fig. 4a-b). In 2D culture, the viability and expansion of NK92 cells decreased sharply after 5 d, whereas in the pore-forming hydrogel, expansion was maintained for up to 7 d [18]. NK92 cells are known to increase cell viability and proliferation by forming clusters that induce cell-to-cell contact signaling [18, 41]. Therefore, NK92 cells encapsulated in the micropore-forming hydrogel ALG/GEL-2 proliferated because cell aggregation actively occurred in the hydrogel with relatively large micropores. Proliferation was similar in ALG and ALG/GEL-1, which can be considered because there was no significant difference in hydrogel properties such as pore size and shrinkage. ALG/GEL-3 showed the lowest proliferation rate because the pore size was too large and cell loss occurred. Therefore, in a subsequent experiment, ALG, which did not intentionally form micropores, and ALG/GEL-2, which could help the viability and proliferation of NK cells, were compared (Fig. 4c).

### Functional characterization of the NK92 cells 2D cultures compared with 3D pore-forming hydrogel

**Fig. 5:**
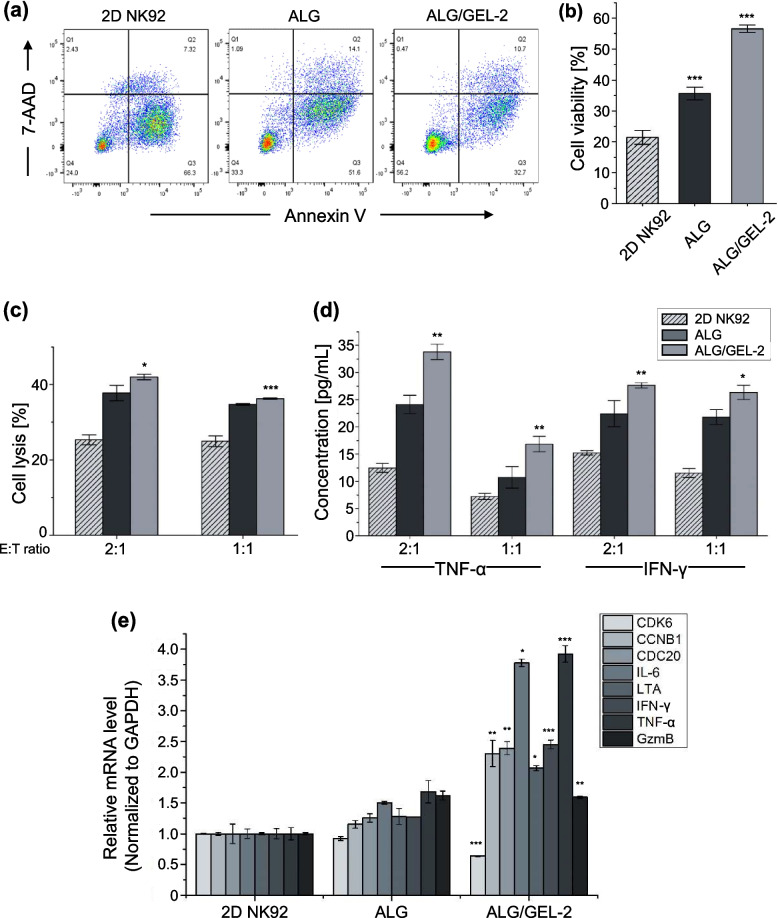
Functional characterization of NK92 2D cultures compared with 3D pore-forming hydrogel after 5 days for cell expansion. **a** Representative flow cytometric data of cell apoptosis and cell death stained by Annexin V and 7-AAD. **b** Bar graph of cell viability from flow cytometric data. **c** Cytotoxicity data of NK92 cell using a Calcein AM-based cytotoxicity assay at different ratio of effector (NK92) to target (K562) (E:T). **d** Cytokine level of TNF-α and IFN-γ by ELISA. **e** mRNA expression level of NK92 by qRT-PCR. [*n* = 3, NS: no significant difference, *p < 0.05, **p < 0.01 and ***p < 0.001]

To use NK92 cells cultured in micro/macropore hydrogels for immunotherapy, the improvement of cell survival and activity in extreme environments is a very important factor. Annexin-V/7-AAD staining was used to observe apoptosis and necrosis to investigate the effect of the pore-forming hydrogel on cell survival (Fig. 5a). After 5 d, the survival rate of 3D cultured NK92 cell, especially in the hydrogel with micropores, was 1.65 - 2.64 times higher than that in the suspension culture group (Fig. 5b).

The cytotoxicity of NK92 cells was investigated at different ratios of effector (NK92) and target (K562) cells (Fig. 5c). NK92 cells were derived from live cells. Higher values of cell lysis were observed in 3D cultures than in 2D cultures. NK cells cultured in ALG/GEL-2 showed the highest lytic activity.

Cytokine secretion of tumor necrosis factor – alpha (TNF-α) and interferon-gamma (IFN-γ) from 3D cultured NK cell was investigated, which is closely related to the cytotoxic function of NK92 cells [42]. The results of the enzyme-linked immunosorbent assay (ELISA) confirmed that the highest cytokine release occurred in NK92 cells in ALG/GEL-2 (1.81 – 2.28 fold) (Fig. 5d).

Moreover, ribonucleic acid (RNA) was extracted from 2 and 3D cultured NK92 cells to compare upregulated genes (Fig. 5e). CDK6, CCNB1, and CDC20, which are related to cell division and proliferation, generally increased in 3D cultures, IL-6, LTA, and TNF-α related to the promotion of the pro-inflammatory signaling response. The expression of IFN-γ and granzyme B (GzmB) was also higher in the 3D cultured group in the micropore-forming hydrogel, which enhanced the cytotoxicity of NK cells and the extinction of the tumor [18]. Therefore, compared with 2D suspension cells, 3D cultures in micro/macropore-forming hydrogels maintained the viability and tumor lytic activity of NK cells. The use of a pore-forming hydrogel provides advantages for the antitumor efficacy of immunotherapy in the tumor microenvironment.

### Anti-tumor effect of the zEGFR-CAR-NK in micro/macropore-forming hydrogel

After the solid tumor is surgically removed, it is a big concern that the remaining tumor cells may threaten the life of a patient [17]. To use immunotherapy for targeting solid tumors, cell expansion with high viability and direct insertion into the tumor site should be solved [17, 18]. In particular, this study aimed to perform cell-based immunotherapy through 3D printing of pore- forming hydrogels to expand immune cells and target the remaining macro-sized tumors. It was investigated as a tumor cell model in vitro to confirm the applicability of 3D bioprinting with a pore-forming hydrogel. Solid tumor-reactive NK cells genetically modified to express chimeric antigen receptors (CARs) composed of zEGFR affibodies were used in this study [18]. CAR-NK is particularly suitable for inducing the recognition and lysis of tumor cells overexpressing EGFR on various tumor cell surfaces, such as breast, lung, and carcinoma [43]. MDA-MB-231 cells expressing EGFR were used as target tumor cells.

**Fig. 6:**
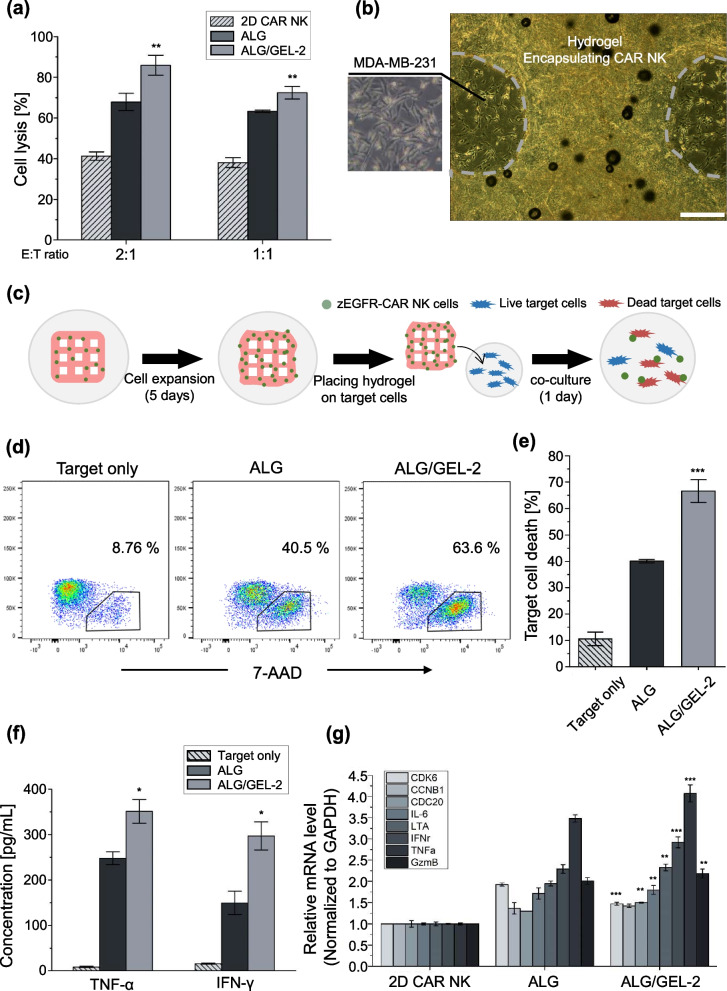
Anti-tumor effect on the zEGFR-CAR-NK cells in micro/macropore-forming hydrogel. **a** Cytotoxicity of CAR-NK cells with different effector to target cell by calcein AM-based assay. **b** Representative optical image of pore-forming hydrogel with CAR-NK cells placed on the MDA-MB-231 target cell, scale bar = 500 μm. **c** Experimental scheme of releasing zEGFR-CAR-NK activity. **d** Representative flow cytometric data of MDA-MB-321 cell death stained by 7-AAD. **e** Target cell death of target only and hydrogel groups from flow cytometric data. **f** Cytokine releasing level of TNF-α and IFN-γ in the cell supernatant by ELISA. **g** mRNA expression level of CAR NK cells by qRT-PCR. [*n* = 3, NS: no significant difference, *p < 0.05, **p < 0.01 and ***p < 0.001]

Cytotoxicity showed a relatively high value compared to NK92 cells (41.9% to 85.9% in ALG/GEL-2 based on E:T = 2:1) because CAR-NK cells specifically induced lysis in target cells. Compared to the suspension culture group, it was confirmed that the cell lysis ability of zEGFR-CAR-NK was approximately 1.66—2.08 times higher, and the micropore group showed higher cytotoxicity (Fig. 6a). Based on these results, the following in vitro model was developed to simulate the antitumor activity when hydrogels including zEGFR-CAR NK cells were implanted at the tumor site. Hydrogels were fabricated by bioprinting and incubated for 5 d to expand and maintain the properties of CAR-NK 3D cultures. When the hydrogels were crosslinked with 3% CaCl_2_, it was confirmed that alginate was naturally degraded by cell metabolism, and NK cells came out after approximately 5 d (Supplementary Fig. 4). The cultured hydrogels were then placed onto the target cells attached to the 24 well plate for 24 h (Fig. 6b-c). The hydrogels and target cells were then co-cultured and target cell lysis was investigated. Judging from the ratio of 7-AAD staining-positive target cells, ALG/GEL-2 showed an approximately 1.66 times higher tumor lytic ability than that of ALG (Fig. 6d-e). The ELISA results also showed that the concentrations of TNF-α and IFN-γ were elevated according to micropore formation (Fig. 6f). The regulation of zEGFR-CAR-NK cell gene expression in the pore-forming hydrogel was confirmed by qRT-PCR. The mRNA expression level changed in 3D cultured CAR-NK cells (Fig. 6g). ALG/GEL-2 showed higher expression levels related to proliferation, cytokine receptor interaction, and NK cell-mediated cytotoxicity. In addition, NK cells induced cell lysis by balancing the mechanisms of activating and inhibitory receptors [44–46]. Among the activating receptors, we confirmed that there was a difference in the expression levels of NKG2D and NKp30. NKG2D is known to mediate direct responses against cellular threats by stimulating the production of cytokines and cytokine molecules, which recognize various ligands upregulated upon cellular stress [44, 47, 48]. The percentages of NKG2D and NKp30 positive cells in ALG/GEL-2 were higher than those in ALG (Supplementary Fig. 5). These results confirmed the anti-tumor efficiency of this in vitro model when the printed hydrogel with immune cells was directly loaded on the tumor site. The cell lytic ability was enhanced in the pore-forming hydrogel.

## Discussion

We demonstrated that the hydrogel encapsulating NK cells created an appropriate micro–macro environment for clinical applications of NK cell therapy for both leukemia and solid tumors via 3D bioprinting. NK cells are highly valued in cell-based immunotherapy due to their low incidence of graft-versus-host disease and relatively low side effects such as on-target off-tumor effect and cytokine release syndrome [4, 8]. Compared to other immune cells such as T cells and B cells, NK cells have the greatest potential as an off-the-shelf product due to their cost-effectiveness and lower side effects [9, 10, 13]. However, accurate delivery of NK cells to the site and maintaining their expansion and cytotoxicity is challenging [8, 14, 15]. By using 3D bioprinting, NK cells cultured in 3D can be produced through an automated system and create macropores, which enhance their viability. To ensure stable micropore formation after printing, a material that can stably form micropores was utilized [22].

The hydrogel forming micropore was controlled by the ratio of alginate and gelatin. Micropore was formed as it is released during incubation. As the gelatin ratio increased, the micropore size also tended to increase. This enables NK cells to form cell clusters, providing an environment favorable for cell viability and proliferation [23]. The forming clusters can induce cell-to-cell contact signaling [18, 40]. Therefore, NK92 cells encapsulated in the micropore-forming hydrogel ALG/GEL-2 proliferated because cell aggregation actively occurred in the ALG/GEL-2 hydrogel with relatively large micropores.

The cytotoxicity and functionality of NK92 cells was investigated in micropore-macropore forming hydrogel. NK cells cultured in ALG/GEL-2 showed the highest lytic activity. Cytokine secretion and mRNA expression were also enhanced in ALG/GEL-2. Therefore, compared with 2D suspension cells, 3D cultures in micro/macropore-forming hydrogels maintained the viability and tumor lytic activity of NK cells [18]. Solid tumor-reactive NK cells were genetically modified to express chimeric antigen receptors (CARs) composed of zEGFR affibodies [17, 18]. CAR-NK is particularly effective at inducing the recognition and lysis of tumor cells that overexpress EGFR on various tumor cell surfaces, such as breast, lung, and carcinoma [43]. The target tumor cells used were breast cancer cells that expressed EGFR. The zEGFR-CAR NK cells cultured in micro/macropore-forming hydrogels confirmed that alginate was naturally degraded by cell metabolism, and NK cells came out after approximately 5 d. The zEGFR-CAR NK cells were released to the target cell and occur cell lysis. And ALG/GEL-2 showed the highest expression levels related to proliferation, cytokine receptor interaction, and CAR-NK cell-mediated cytotoxicity. Furthermore, NK cells induce cell lysis by balancing the mechanisms of activating and inhibitory receptors [44–46]. Among the activating receptors, we observed differences in the expression levels of NKG2D and NKp30. These receptors are known to mediate direct responses against cellular threats by stimulating the production of cytokines and cytokine molecules that recognize various ligands upregulated upon cellular stress [44, 47–49]. The NKG2D and NKp30 were upregulated in ALG/GEL-2. This results showed that the in vitro model using a printed hydrogel loaded with immune cells exhibited effective anti-tumor activity when directly applied to the tumor site.

## Conclusions

Two major issues emerging in immunotherapy are cell expansion and the direct loading of immune cells into a target tumor. In this study, a micro/macropore-forming hydrogel with immune cells was designed for cell expansion with suitable viability by facilitating cell-to-cell interactions, and 3D bioprinting was introduced by direct loading on the tumor site. To confirm the possibility of immunotherapy, the characteristics of pore-forming hydrogel, expansion with viability of immune cells in 3D culture, and functionality with an in vitro model were investigated. By investigating the activity of NK92 and zEGFR-CAR-NK cells as leukemia and solid tumor cells, the possibility of their application to various types of tumors was confirmed. Bioinks with suitable shear-thinning and rheological properties for printing were fabricated by adjusting the ratio of alginate to gelatin. It was confirmed that the micropores formed as gelatin were released and had printability and shrinkage characteristics. Cell viability, cell clusters, and proliferation were compared between the groups with different micropore sizes. As ALG/GEL-2 showed the highest viability and proliferation, it was used in subsequent in vitro experiments. In comparison with NK92 cells cultured in 2D and 3D hydrogels, the pore-forming hydrogel could enhance the cell condition and activities such as viability, cytotoxicity, and cytokine release. It was also suggested that zEGFR-CAR NK cells released from the hydrogel could effectively cause lysis of the target cell when implanted at the tumor site, especially for solid tumors. Cytotoxicity, cytokine secretion, the expression of activating receptors and mRNA expression levels related to proliferation, pro-inflammatory signaling response, and cytotoxicity of pore-forming hydrogels were higher than those of ALG. These results imply that this system could overcome the obstacles of conventional delivery systems, which are related to the expansion and localization of immunotherapy. Pore-forming hydrogels with immune cells can be used in clinical applications to prevent tumor recurrence and metastasis after tumor resection.

## Supplementary Information

**Additional file 1: Supplementary Fig. 1.** Comparison of proliferation, viability and cytotoxicity according to NK92 cell density cultured in two-dimensions. (a) Cell number of NK92 cell according to density. The higher the cell density, the higher the rate of increase. (b) Cell proliferation folds of NK92 cell. (c) Cell apoptosis and death on the day 5. (d) NK92 cell lytic activity based on Calcein-AM cytotoxicity assay on day 3 and 5 at and E:T ratio of 2:1 and 1:1. **Supplementary Fig. 2.** Printing conditions of ALG and ALG/GEL-2 groups related to pressure and printing feed rate. ALG groups were printed at 300 mm/min at 60 kPa of pressure and ALG/GEL-2 group were printed at 300 mm/min at 70 kPa. Scale bar = 400 µm. **Supplementary Fig. 3.** EDTA concentration test for cell viability and alginate degradation. (a) Representative microscopic image of formation of NK92 cell clusters and viability related to cell condition according to EDTA concentrations. NK92 cells tend not to maintain cell clusters over 8mM. Scale bar = 100 µm. (b) Degradation of 3% alginate hydrogel for 3D encapsulated NK92 cells preparation. 8 mM EDTA concentration were used for hydrogel degradation. Scale bar = 1mm. **Supplementary Fig. 4.** Concentration of calcium chloride for alginate crosslinking. When ALG and ALG/GEL-2 groups were crosslinked by 3% CaCl_2_, NK92 cells were released out by hydrogel degradation on day 5. Scale bar = 500 µm. **Supplementary Fig. 5.** Expression of activating receptors at 3D cultured zEGFR-CAR NK cells. **Supplementary Table 1.** Gene specific primer sequences for qRT-PCR.

## Data Availability

All data associated with this study are present in the paper or the Supplementary information. All relevant data are available from the authors.
