# Supplementary material for: NK cells encapsulated in micro/macropore-forming hydrogels via 3D bioprinting for tumor immunotherapy
Source: Biomater Res. 2023 Jun 22;27:60. doi: 10.1186/s40824-023-00403-9 (PMC10286468; doi:10.1186/s40824-023-00403-9)

**NK cells encapsulated in micro/macropore-forming hydrogel via 3D bioprinting for tumor immunotherapy**

Dahong Kim, Seona Jo, DongJin Lee, Seok-Min Kim, Ji Min Seok, Seon Ju Yeo, Jun Hee Lee, Jae Jong Lee, Kangwon Lee, Tae-Don Kim^†^, and Su A Park^†^


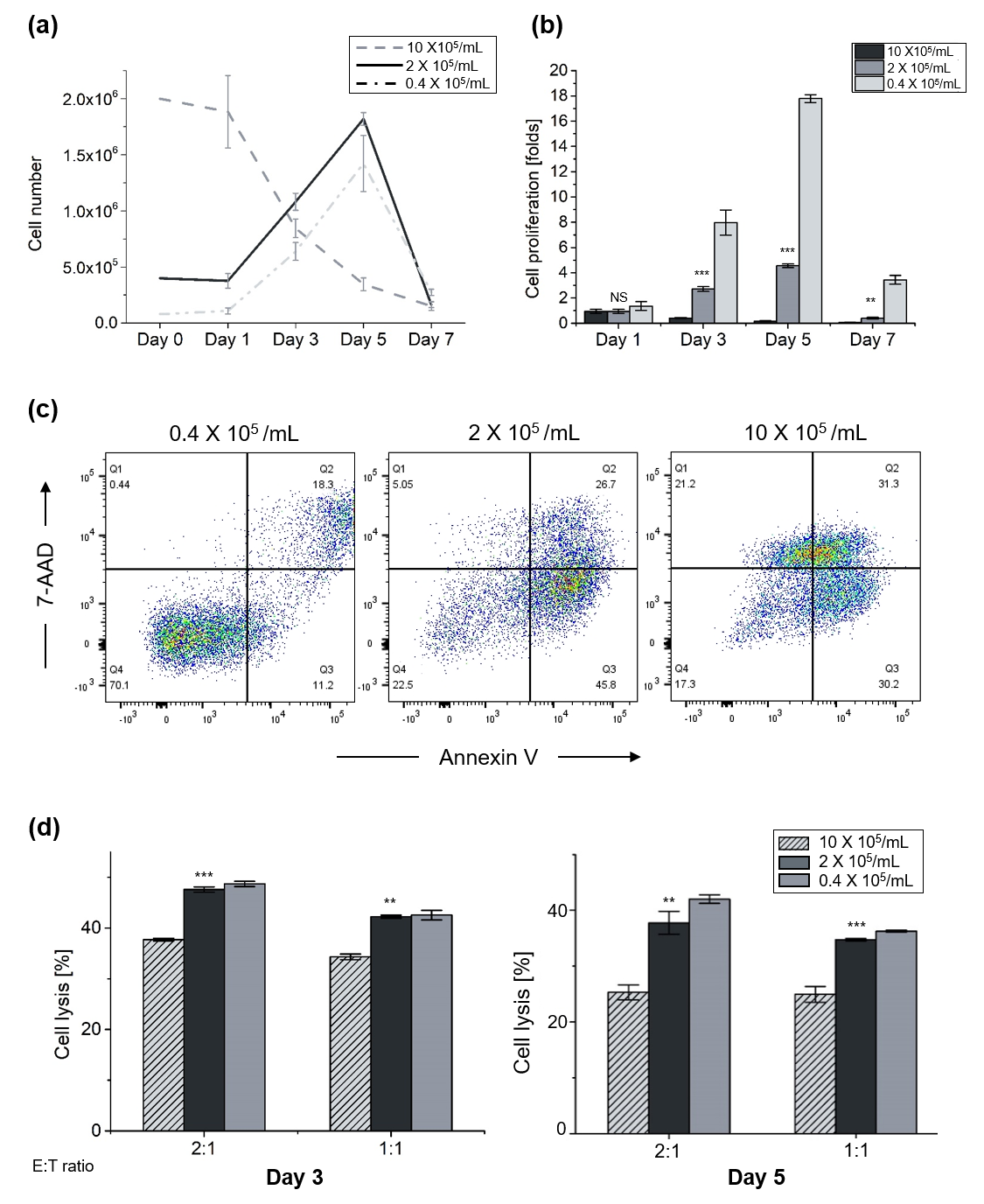


**Supplementary Fig. 1** Comparison of proliferation, viability and cytotoxicity according to NK92 cell density cultured in two-dimensions. **(a)** Cell number of NK92 cell according to density. The higher the cell density, the higher the rate of increase. **(b)** Cell proliferation folds of NK92 cell. **(c)** Cell apoptosis and death on the day 5. **(d)** NK92 cell lytic activity based on Calcein-AM cytotoxicity assay on day 3 and 5 at and E:T ratio of 2:1 and 1:1.


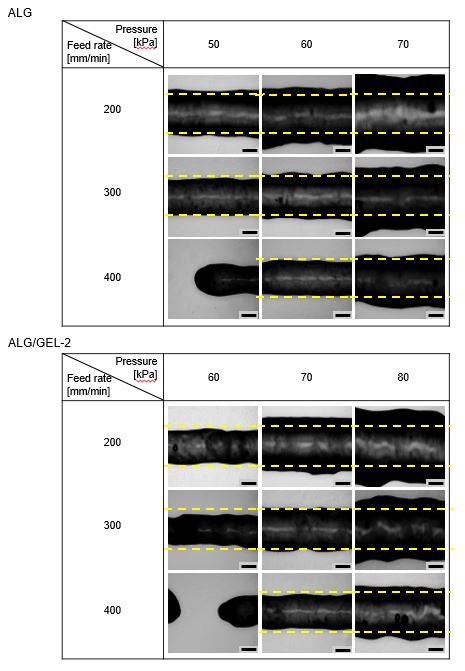


**Supplementary Fig. 2** Printing conditions of ALG and ALG/GEL-2 groups related to pressure and printing feed rate. ALG groups were printed at 300 mm/min at 60 kPa of pressure and ALG/GEL-2 group were printed at 300 mm/min at 70 kPa. Scale bar = 400 µm.


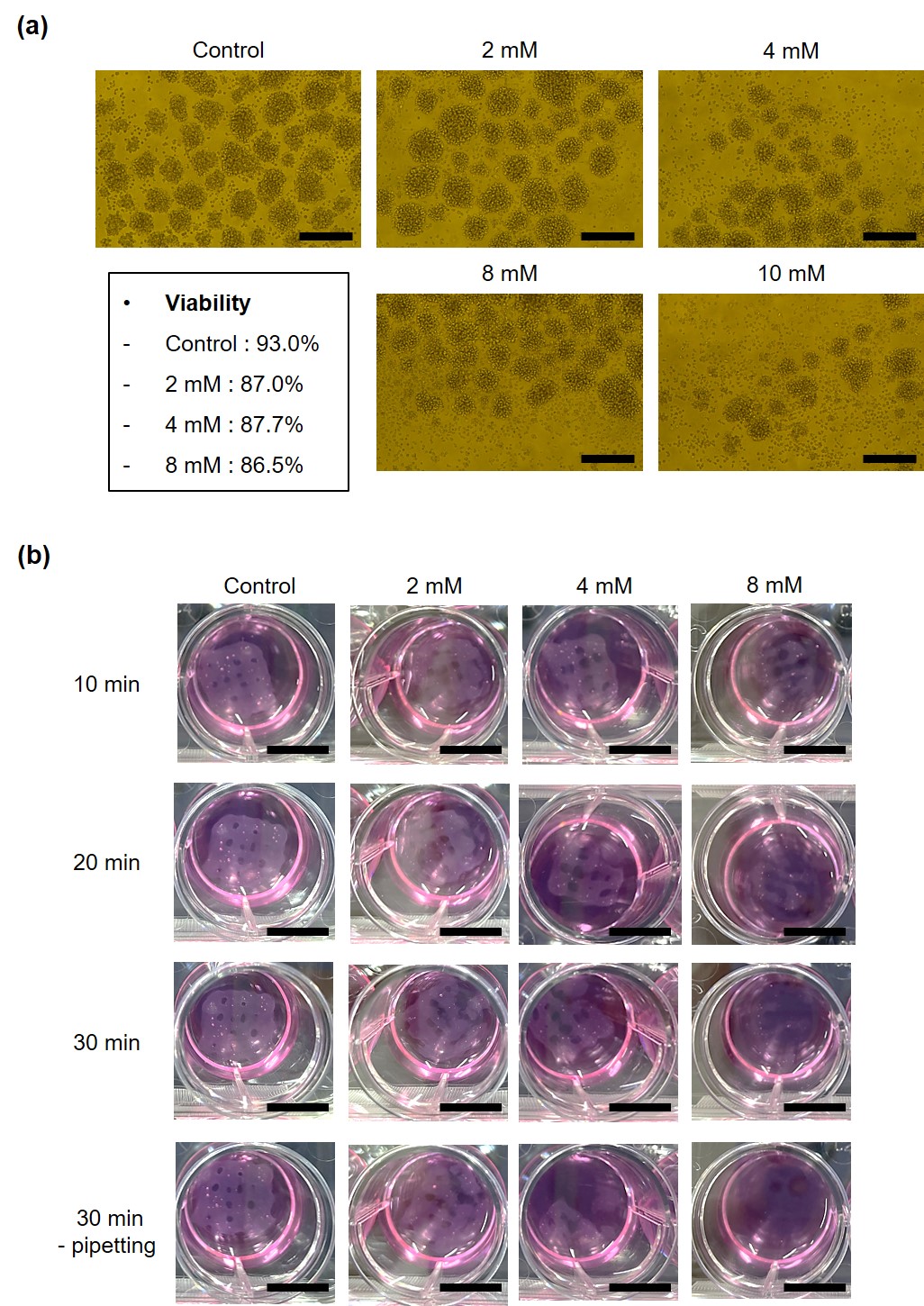


**Supplementary Fig. 3** EDTA concentration test for cell viability and alginate degradation. **(a)** Representative microscopic image of formation of NK92 cell clusters and viability related to cell condition according to EDTA concentrations. NK92 cells tend not to maintain cell clusters over 8mM. Scale bar = 100 µm. **(b)** Degradation of 3% alginate hydrogel for 3D encapsulated NK92 cells preparation. 8 mM EDTA concentration were used for hydrogel degradation. Scale bar = 1mm.


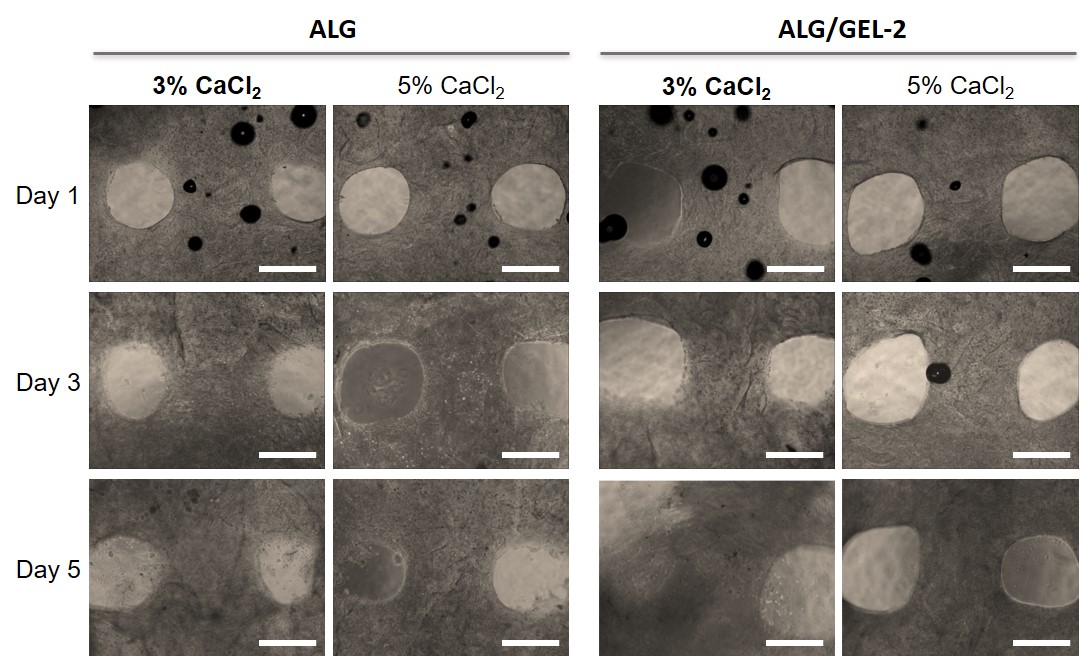


**Supplementary Fig. 4** Concentration of calcium chloride for alginate crosslinking. When ALG and ALG/GEL-2 groups were crosslinked by 3% CaCl_2_, NK92 cells were released out by hydrogel degradation on day 5. Scale bar = 500 µm.


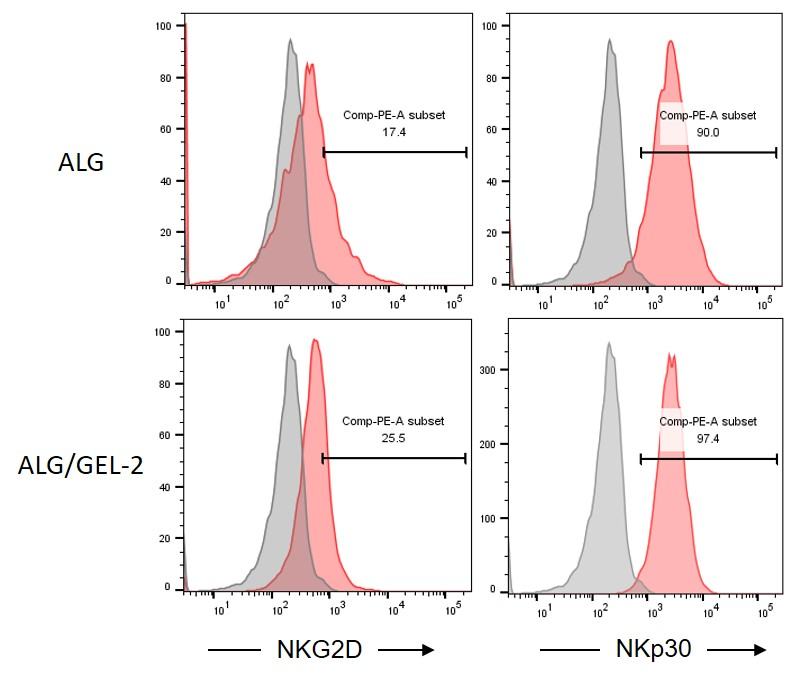


**Supplementary Fig. 5** Expression of activating receptors at 3D cultured zEGFR-CAR NK cells (gray: 2D cultured zEGFR-CAR NK cell, red: 3D cultured zEGFR-CAR NK cell).

**Supplementary Table 1.** Gene specific primer sequences for qRT-PCR.


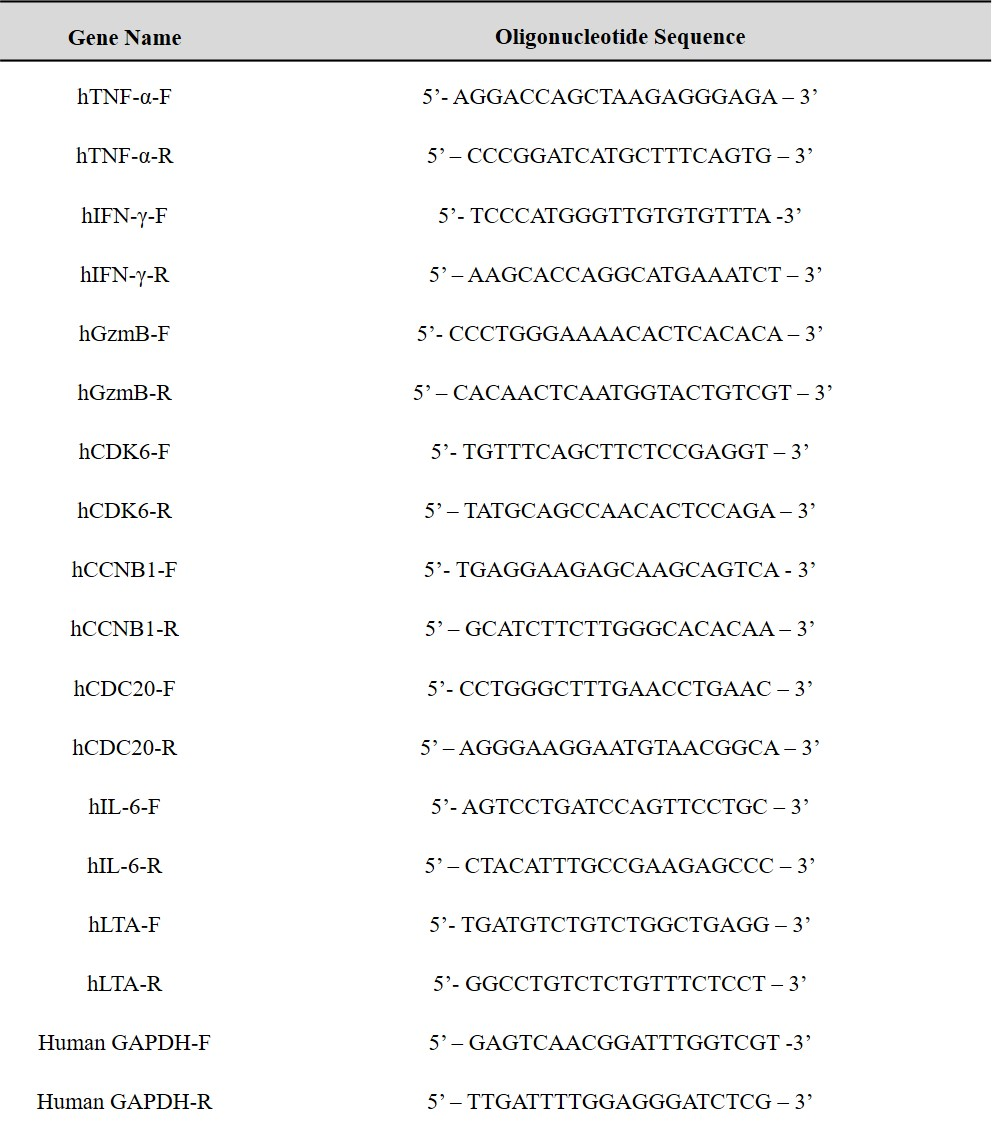

Supplement: Supplementary file 1 — Additional file 1: Supplementary Fig. 1. Comparison of proliferation, viability and cytotoxicity according to NK92 cell density cultured in two-dimensions. (a) Cell number of NK92 cell according to density. The higher the cell density, the higher the rate of increase. (b) Cell proliferation folds of NK92 cell. (c) Cell apoptosis and death on the day 5. (d) NK92 cell lytic activity based on Calcein-AM cytotoxicity assay on day 3 and 5 at and E:T ratio of 2:1 and 1:1. Supplementary Fig. 2. Printing conditions of ALG and ALG/GEL-2 groups related to pressure and printing feed rate. ALG groups were printed at 300 mm/min at 60 kPa of pressure and ALG/GEL-2 group were printed at 300 mm/min at 70 kPa. Scale bar = 400 µm. Supplementary Fig. 3. EDTA concentration test for cell viability and alginate degradation. (a) Representative microscopic image of formation of NK92 cell clusters and viability related to cell condition according to EDTA concentrations. NK92 cells tend not to maintain cell clusters over 8mM. Scale bar = 100 µm. (b) Degradation of 3% alginate hydrogel for 3D encapsulated NK92 cells preparation. 8 mM EDTA concentration were used for hydrogel degradation. Scale bar = 1mm. Supplementary Fig. 4. Concentration of calcium chloride for alginate crosslinking. When ALG and ALG/GEL-2 groups were crosslinked by 3% CaCl2, NK92 cells were released out by hydrogel degradation on day 5. Scale bar = 500 µm. Supplementary Fig. 5. Expression of activating receptors at 3D cultured zEGFR-CAR NK cells. Supplementary Table 1. Gene specific primer sequences for qRT-PCR. [file 40824_2023_403_MOESM1_ESM.docx]
